# Pectinolytic Bacterial Consortia Reduce Jute Retting Period and Improve Fibre Quality

**DOI:** 10.1038/s41598-020-61898-z

**Published:** 2020-03-20

**Authors:** Rajnee Hasan, Nasima Aktar, Shah Md. Tamim Kabir, Ummay Honi, Abdul Halim, Rahin Islam, Muhammad Delwar Hossain Sarker, Md. Samiul Haque, Md. Monjurul Alam, Md. Shahidul Islam

**Affiliations:** 1 Basic and Applied Research on Jute Project, Bangladesh Jute Research Institute, Dhaka, Bangladesh; 2 McMaster University, Hamilton, Canada; 3 Bangladesh Jute Research Institute, Dhaka, Bangladesh; 4 Eskayef Pharmaceuticals Limited, Dhaka, Bangladesh

**Keywords:** Environmental biotechnology, Applied microbiology

## Abstract

Jute fibre is the second most important fibre next to cotton. It is obtained from the bark of plant through microbial retting process. Here we report optimized microbial retting protocol that can lower retting period and produce high fibre quality. A total of 451 bacterial colonies have been isolated from five jute retting water samples in Bangladesh. Higher pectinolytic bacterial isolates were predominant in the later stage of jute retting. Out of these, 168 isolates have been screened by both semi-quantitative and quantitative pectinase, xylanase and cellulase enzyme assay. Among them, 144 isolates have been selected on the basis of extra cellular enzyme activity of these three enzymes. 16 s ribosomal gene sequencing analysis identified 2 phyla- *Firmicutis* (80.55%) and *Proteobacteria* (19.45%). To check the synergistic and antagonistic effect 10 selected isolates were tested in 167 different combinations. Three best combinations were identified that lowered retting period from 18–21 days to 10 days producing high quality fibre in both laboratory and field trial. This improved retting technology can be adopted in industrial scale for the production of quality jute fibre in a controlled condition in reduced water quantity without polluting the environment.

## Introduction

Jute, second most important natural fibre after cotton, is cultivated in East Asia and some parts of Latin America^1–3^. Jute is a bast or phloem fibre in the bark of stems, cemented together by pectin and gummy substances^4^. Commercial extraction of jute fibre is water based microbiological retting where jute bundles are submerged into slow running river water and subjected to decomposition of pectin, hemicelluloses, and other mucilaginous substances^2,5^. In this process, pectin is depolymerized by pectinases, primarily comprising four enzymes: Polygalacturonase (PG), Pectin Lyase (PNL), Pectate lyase and Pectin esterase. However, PG^6^, and PNL^7^ are primary retting enzymes. In addition, xylanase makes jute fibre softer by selective removal of non-fibrous hemicelluloses without affecting strength of cellulosic fibre. Pectinolytic microorganisms having xylanase activity devoid of cellulase activity is an additional beneficial aspect to improve fibre quality^8^. The quality of fibre is largely determined by the efficiency of retting process^1,2,9,10^ and various factors are responsible for proper retting as well as improved fibre quality. Most promising water based microbiological retting process mostly involves bacteria along with various fungi, protozoa, algae and diatoms^10–13^. Main aerobic retting bacteria belong to genus *Bacillus* viz., *B. subtilis, B. polymyxa, B. mesentericus, B.pumilus, B. cereus, B. megaterium and B. macerans*, initiate retting^14–18^ along with large numbers of gram-negative genera such as *Erwinia* and *Pseudomonas*^9,19^. At the later stage of retting some anaerobic bacteria from genus *Clostridium - Clostridium acetobutylicum, Clostridium stercorarium*, *Clostridium tertium* come to carry on the retting process^20^. As microorganisms are the main pectinolytic agent during retting, affecting the process and end product quality in depth knowledge of microbial community is essential. Moreover, retting water varies from place to place with respect to its physico-chemical, microbial and biochemical properties which affect fibre quality^21,22^. Due to scarcity of water, farmers are compelled to use water bodies repeatedly for retting, leading to poor quality jute fibre production^21,23^. Acute shortage of water and the environmental pollution created from conventional system of retting has demanded for improving the retting process. Aim of this work was to optimize microbial retting protocol that will lower retting period and increase fibre quality. Our strategy involved analysis of pectinolytic retting bacteria with selection of best synergistic effect producing combinations of microbes. In summary, we had to optimize retting process with the best bacterial consortium to yield high quality fibre with less volume of water by reducing retting time from 18–21 days to 10 days.

## Results and Discussion

### Isolation and screening of isolates

**Figure 1:**
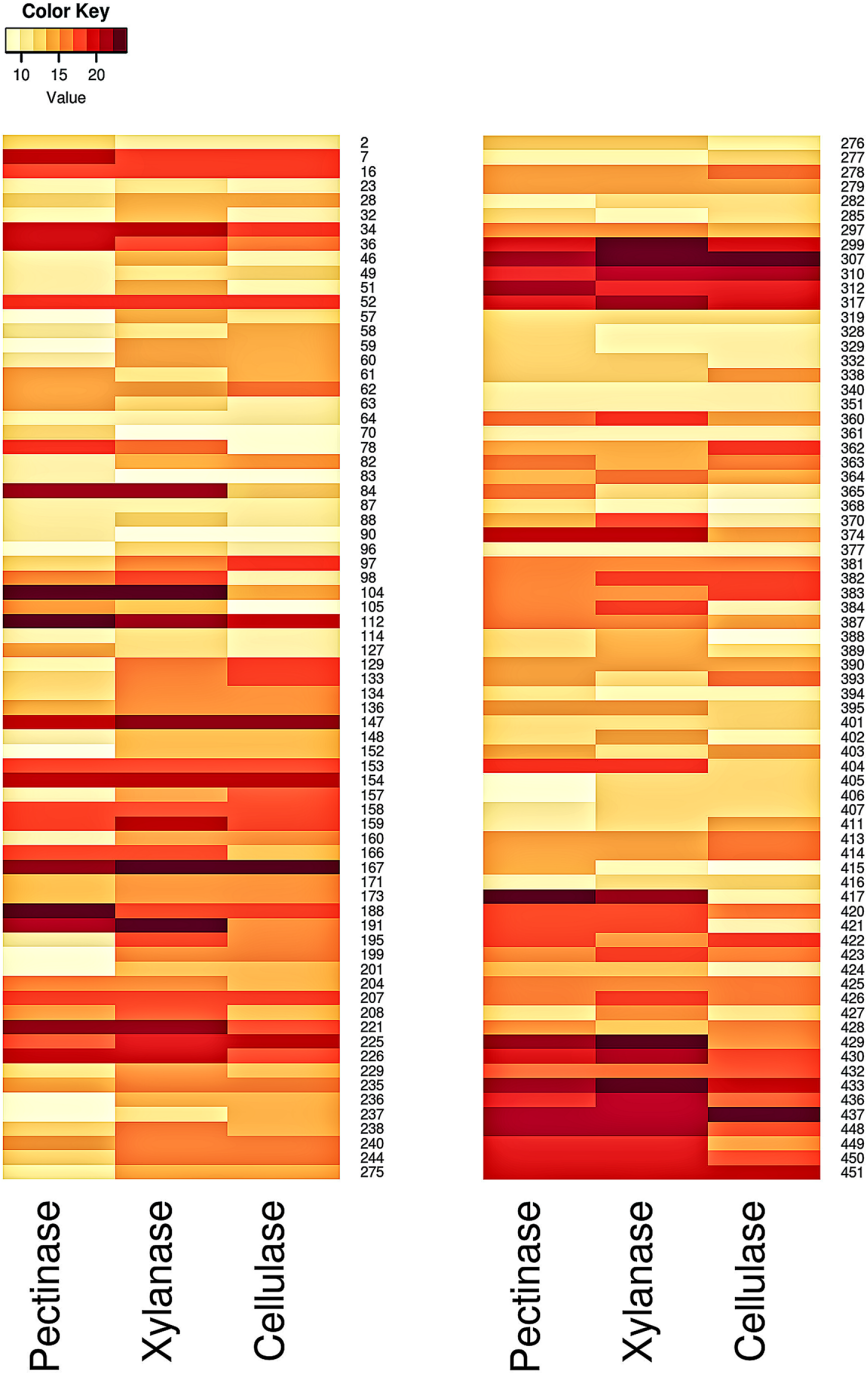
Heat map of liquid culture extra cellular pectinase, xylanase and cellulase activity of 144 isolates. Yellow to red color indicates lowest to highest enzyme activity.

Four hundred and fifty one bacterial isolates were selected based on the colony morphology (Fig. S1). Most of the isolates were found to produce cream color colonies with dry or moist surface. Majority of them were rod shaped and spore former. However some colonies with different color and shape were also observed (Fig. S1). The colony morphology of isolates, as found here in, is comparable with the previous report by Das *et al*.^3^. Qualitatively these bacteria were screened on the basis of zone of hydrolysis around the bacterial growth. Out of 451 isolates, 168 having high pectinolytic and xylanolytic and low or no cellulolytic activities were selected (Supplementary Table 1). In primary screening, the colonies forming clear zone, after flooding the substrate with iodine solution indicated to be the enzyme producers (Fig. S2). Efficiency of these isolates was further assessed for extra cellular enzymatic activities through liquid culture (Fig. S3). Finally 144 potential isolates were obtained as pectinase, xylanase and low cellulase producer by forming clear zone within the range of 7.5–25.5 mm, 8.5–25.5 mm and 7.0–25.0 mm diameter respectively (Supplementary Table 2 and Fig. 1). In this study, microorganisms with high pectinolytic, xylanolytic and low cellulolytic activities have been focused because quality of jute fibre mostly depends on retting process and the retting process mainly depends on these enzymatic activities of microorganisms^9,15,17^. Polygalacturonase is considered to be the most important enzyme in this process^6,24,25^ and xylanase is required for the partial removal of hemicellulose that makes the jute fibre softer for finer spinning^26,27^. So it is prime need to have these enzymatic activities of isolates for efficient use as retting inocula.

### Identification and phylogenic analysis

**Figure 2:**
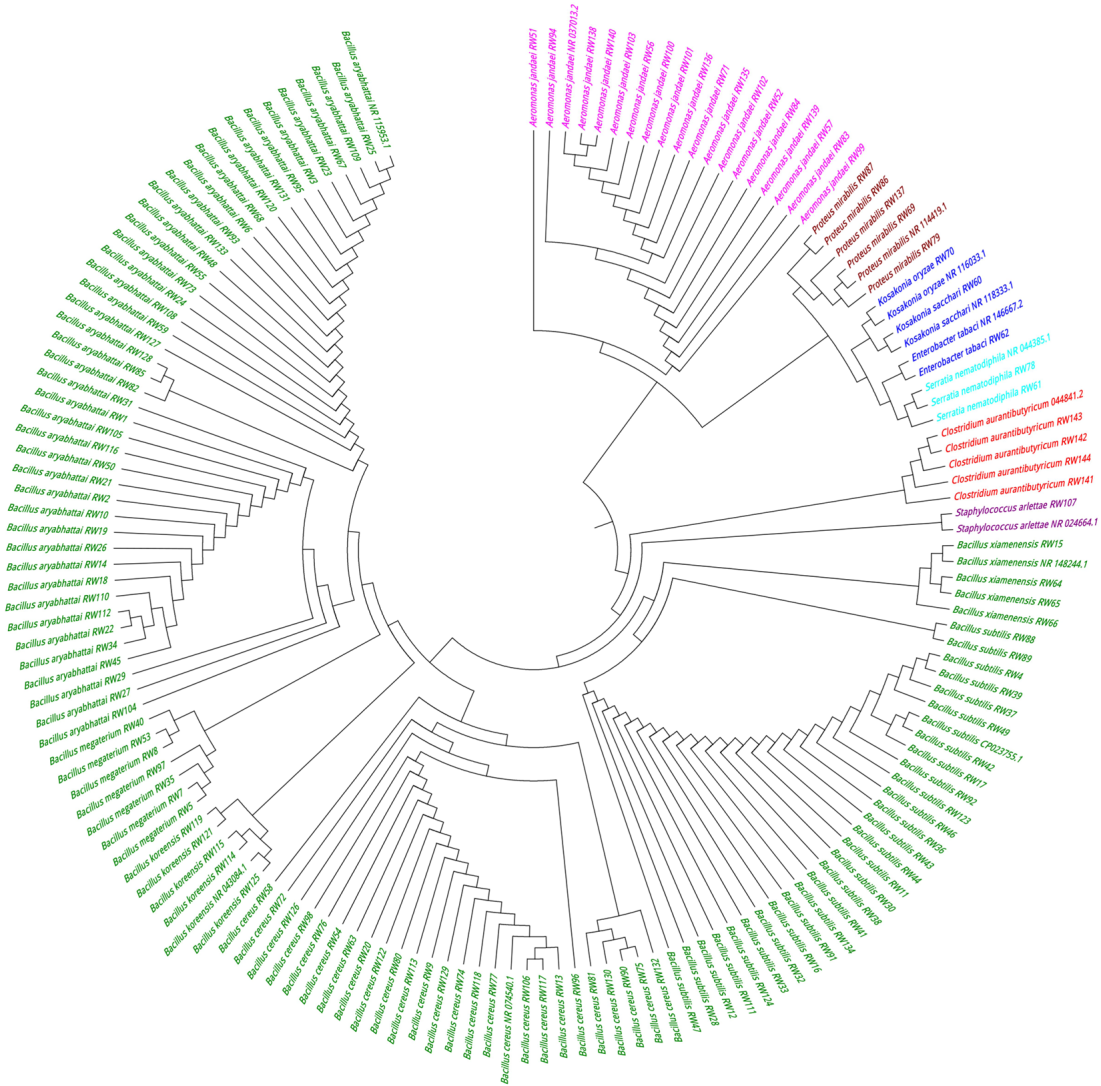
Neighbor-joining phylogenetic tree construction using 16S rRNA gene sequences by Mega 6.0.

**Figure 3:**
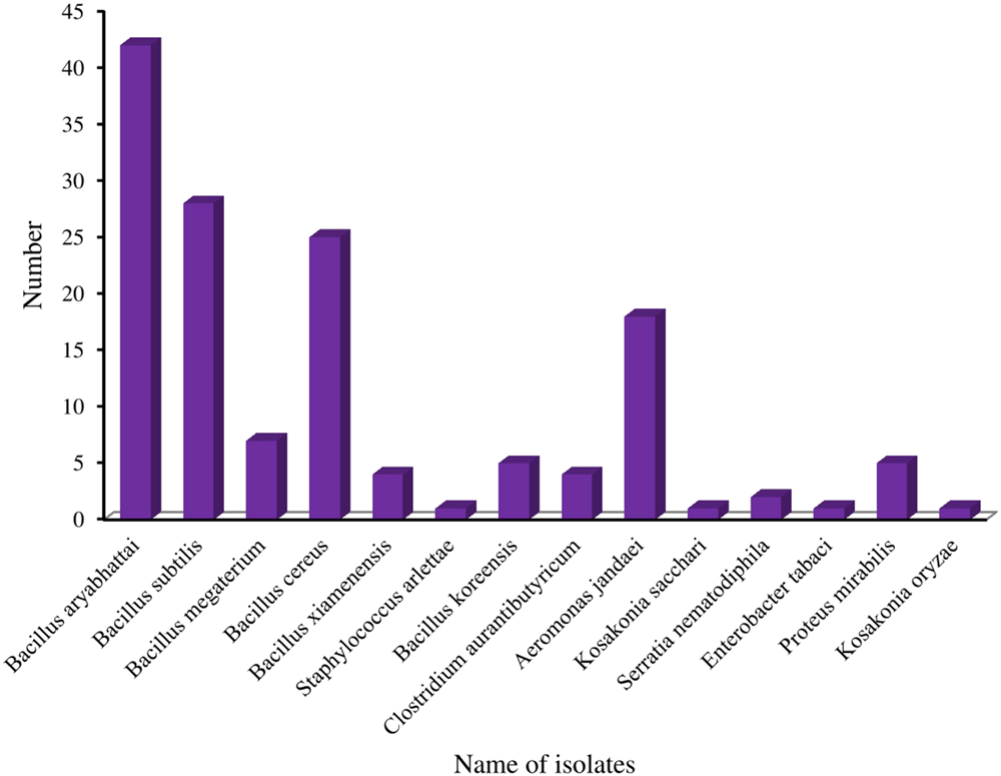
Relative distribution of 14 species found in the 16S rRNA gene sequences analysis of jute-retting water.

A molecular approach based on 16S rRNA gene sequencing was used to identify and distinguish closely related bacterial strains (Supplementary Table 3) and the retrieved sequences were used to construct phylogenetic trees to show relative positions of the isolates at genus level. The 16s rDNA sequence analysis and estimation of phylogenetic relationships (Fig. 2) assigned all 144 strains into two predominant phyla-*Firmicutes* (most abundant components ~80.55%) and *Proteobacteria* (~19.45%) which corroborated with the findings of Munshi and Chattoo^2^. The comparative sequence analysis revealed a rich spectrum of bacterial diversity. The total of 144 bacterial isolates belonged to 14 phylogenetically related species- *Bacillus aryabhattai* (29%), *Bacillus subtilis* (19.44%), *Bacillus cereus* (17.36%), *Bacillus megaterium* (4.86%), *Bacillus koreensis* (3.47%), *Bacillus xiamenensis* (2.78%), *Staphylococcus arlettae* (0.69%), *Clostridium aurantibutyricum* (2.78%), *Aeromonas jandaei* (12.5%), *Proteus mirabilis* (3.47%), *Serratia nematodiphila* (1.39%), *Kosakonia sacchari* (0.69%), *Kosakonia oryzae* (0.69%) and *Enterobacter tabaci* (0.69%) under 8 genera (Fig. 3). Comparative sequence analysis revealed that 144 isolates belong to phylogenetically related 14 species and the *Bacillus spp*. was dominant. Many *Bacillus sp*. are also widely reported to produce enzymes of industrial application in paper industry^28–33^ and bast fibre degumming^34,35^. The abundance of these species in our study is in accordance with the earlier culture-based studies, which implicate their role in the retting of jute^3,13^. Interestingly, we have identified seven phylogenetically related bacterial species namely *Aeromonas jandaei, Proteus mirabilis, Bacillus xiamenensis, Bacillus koreensis, Serratia nematodiphila, Kosakonia oryzae* and *Enterobacter tabaci* as retting bacteria which were not reported in previous study as jute retting bacteria.

### Selection of best retting bacterial strains

Out of 144, only 10 bacterial strains were selected from retting niche based on extracellular enzymatic activities for preparing retting consortia (Table 1). The selected 10 bacterial isolates comprised namely *Bacillus megaterium*, *Bacillus subtilis*, *Bacillus cereus*, *Bacillus xiamenensis*, *Bacillus koreensis, Proteus mirabilis, Enterobacter tabaci, Kosakonia oryzae, Serratia nematodiphila* and *Aeromonas jandaei*. Among them, *Bacillus megaterium*, *Bacillus subtilis* and *Bacillus cereus* were previously reported to play role in retting process^2,3,23^. Rest of seven phylogenetically related species, *Bacillus xiamenensis, Aeromonas jandae, Proteus mirabilis, Serratia nematodiphila, Bacillus koreensis, Kosakonia oryzae and Enterobacter tabaci* were not previously studied as retting bacteria. *Bacillus megaterium* showed highest pectinolytic and xylanolytic activity.

**Table 1:** Representative 10 bacterial strains along with their enzymatic activities.

ID	Species	Enzyme Activity					
		Pectinas		Xylanase		Cellulase	
		Zone size (mm)	U/ml	Zone size (mm)	U/ml	Zone size (mm)	U/ml
84	*Bacillus xiamenensis*	21	9.68	21	3.19	13	3.87
221	*Bacillus cereus*	21.5	9.69	21	3.37	15.5	6.73
374	*Bacillus koreensis*	20	9.20	20	2.17	14.5	5.41
417	*Bacillus megaterium*	25	14.59	23	5.80	10.5	1.53
421	*Bacillus subtilis*	19	8.71	20	2.67	10	1.02
78	*Enterobacter tabaci*	18	7.55	16	1.75	9.5	0.82
104	*Proteus mirabilis*	23	11.36	23.5	6.38	14	5.70
105	*Kosakonia oryzae*	16	6.22	15	1.63	8.5	0.61
166	*Serratia nematodiphila*	17	6.87	16	1.87	10.6	0.98
191	*Aeromonas jandaei*	22.5	10.64	23.5	6.03	12.5	2.23
LSD (*P* < 0.05)^a^		0.875	0.217	0.846	0.172	0.894	0.243
Coefficient of Variation (%)		2.98	1.34	2.93	3.10	5.22	4.90

^a^Least significant difference.

### Comparison of individual and consortial enzyme activity

Strains within various species usually differ in their capacity to ret jute due to varying in enzyme activities. From 10 selected isolates, the highest pectinase activity was noted in *Bacillus megaterium* (ID-417; 25 mm clear zone, 14.59 U/ml) while lowest was in *Kosakonia oryzae* (ID-105; 16 mm clear zone, 6.22 U/ml). *Aeromonas jandae* (ID-191; 23.5 mm clear zone, 6.03 U/ml) and *Proteus mirabilis* (ID-104; 23.5 mm clear zone, 6.38 U/ml) recorded as the highest xylanase activity while it was lowest in *Kosakonia oryzae* (ID-105; 15 mm clear zone, 1.63 U/ml) (Table 1). We observed that the pectinolytic and xylanolytic activities associated with cellulolytic activity. Every pectinolytic microbes found in our study had cellulolytic activity. Brühlmann, and his colleague^36^ reported that cellulolytic activities were always found to be associated with pectinolytic and xylanolytic activities.

**Figure 4:**
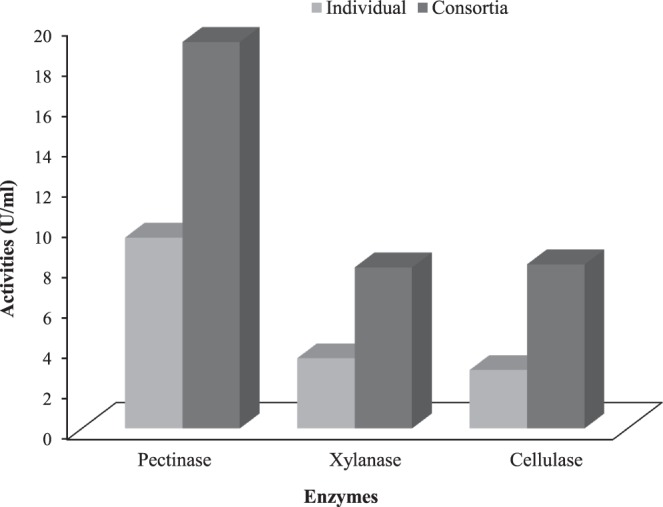
Comparison of mean pectinase, xylanase and cellulose activities of 10 representative individual isolates with 3 selected bacterial consortia.

Based on pectinolytic and xylanolytic activities 10 bacterial isolates were selected for retting consortia. Because, a mixture of microbes secreting different enzymes are more effective for retting of jute plants than a single microbe^37^. Microbial consortia included 10 phylogenetically related species namely, *Bacillus megaterium*, *Bacillus subtilis*, *Bacillus cereus*, *Bacillus xiamenensis*, *Bacillus koreensis, Proteus mirabilis, Enterobacter tabaci, Kosakonia oryzae, Serratia nematodiphila* and *Aeromonas jandaei*. One hundred and sixty seven combinations were evaluated for their enzyme activities (Supplementary Table 4). Finally, three consortia namely C-51, C-67 and C-90 were (having highest pectinolytic and xylanase activities however lower cellulolytic activity) selected as potential for use in large scale jute retting process after screened for antagonism. In most of the cases, all the enzyme activities of the consortia were higher than the individual organisms. For example, consortium C-67 showed higher pectinase (31.5 mm clear zone, 21.4 U/ml) and xylanase (30.5 mm clear zone, 8.56 U/ml) activities (Table 2) corroborating the potentiality of using microbial consortia instead of single microbial strain (Fig. 4).

**Table 2:** Enzyme activities of three selected bacterial consortia.

Combination name	Strains	ID of isolates*	Enzyme Activity					
			Pectinase		Xylanase		Cellulase	
			Zone size (mm)	U/ml	Zone size (mm)	U/ml	Zone size (mm)	U/ml
C-51	5	78, 191, 105, 221, 84	28.0	17.0	27.5	7.67	15.0	6.63
C-67	6	78, 191, 105, 221, 84, 374	31.5	21.4	30.5	8.56	20.0	9.92
C-90	7	78, 191, 105, 221, 84, 421, 417	29.5	19.0	27.25	7.71	16.5	7.84
LSD (*P* < 0.05)^a^			0.998	0.785	0.957	0.399	0.576	0.551
Coefficient of Variation (%)			1.95	1.81	1.93	2.21	1.94	2.98

^a^Least significant difference; *Details are given in Supplementary Table 3.

### Effectiveness of bacterial consortia in retting

Three bacterial consortia, C-51, C-67 and C-90 with an inoculum of 1 × 10^8^ CFU/ml concentrations and a 5:1 water-inocula ratio reduced the retting period to 10–11 days from 21days (Table 3). It also made remarkable improvement in jute fibre strength, twisting force and luster over control. The results showed that the fibre strength, twisting force and luster increased when microbial consortia were used for retting compared with positive control. The increment of fibre strength and luster could amount to about 20% and fibre twisting force could amount to 9%. It indicated that retting with microbial consortia could results in the effectively removal of pectinolytic material linking gum component within a short time from the fibre. It also confirmed that the microbial consortia did not damage jute fibre. Tenkanen and his colleague^38^ have shown that the residual lignin linking with xylan in birch kraft pulp could be removed by xylanase. Higher fibre strength, twisting force and luster could be achieved by using C-67 consortium compared with C-90 and C-51. The results clearly demonstrated that microbial consortia significantly reduced the retting period as well as improved the fibre quality in respect of strength, twisting force and luster. Among them, C-67 consortium gave the best result that reduced the retting time from 18–21days to 10 days with better fibre quality. Thus water-retting process and fibre quality were substantially improved by simultaneously inoculating water tanks with six selected pectinolytic strains. Saha *et al*.^39^ used a microbial consortia for jute retting comprising three strain of *Bacillus pumilus* which retted jute plants in 13–15 days. But in the current study, bacterial consortia comprising more than one species has been used to reduce retting period from 18–21 days to 10 days. It is also worth mentioning here that the bacterial consortium is capable of improving fibre strength, color and fineness without using any chemical.

**Table 3:** Average retting period and quality parameter of jute fiber.

Combination No	Treatment Contents (Water:Culture)	Average Retting Period (Days)	Fiber Strength (lb/mg)	Twisting Force (turns/cm length)	Luster	Fiber Color
C-51	10:1	13	8.57 ± 0.04	8.75 ± 0.14	33.33 ± 0.13	Golden yellow
	5:1	11	9.34 ± 0.13	9.70 ± 0.05	33.97 ± 0.02	Golden yellow
C-67	10:1	12	8.58 ± 0.11	10.08 ± 0.01	34.24 ± 0.09	Golden yellow
	5:1	10	11.06 ± 0.03	10.40 ± 0.11	39.46 ± 0.03	Golden yellow
C-90	10:1	12	8.96 ± 0.02	9.32 ± 0.01	31.92 ± 0.08	Golden yellow
	5:1	11	9.27 ± 0.02	9.59 ± 0.03	32.35 ± 0.02	Golden yellow
Positive control	10 L River water	24	9.1 ± 0.06	9.52 ± 0.08	32.38 ± 0.17	Golden yellow
Negative control	10 L distilled water	26	7.46 ± 0.02	8.96 ± 0.03	21.42 ± 0.21	Golden yellow

## Conclusion

Jute cultivation in water scarce environment is under threat due to higher water requirements for separating fibre from jute stem through retting process. The present study indicated that selective inoculation with efficient microorganisms could be an alternative method of jute retting to obtain quality fibre in water scarce environment. The consortium C-67 could be adopted after commercial field trial to ret jute in minimum water within short time.

## Methods

### Source of plant material

Defoliated 120 days aged green jute stem (*Corchorus olitorius* variety 04) samples were collected from Jute Experimental Research Station, Manikganj, Bangladesh (23°52′N, 90°1.4′E), and sub-merged in slow moving Dhaleswari River Water (DRW).

### Retting sample collection

Five retting liquor samples namely- DRW-1, DRW-2, DRW-3, DRW-4 and DRW-5, were collected at four days interval within 4–20 days of retting from close proximity of submerged mat of jute bundles to get maximum coverage of retting microbial population. Three sub-samples of retting water were collected from evenly spaced parts (upper, middle and lower) of the jute bundle for each sample at a depth of 20 cm from water surface. All the samples were immediately placed on ice for transportation. The three sub-samples were pooled to create a single composite sample and filtered using Cheese cloth (50–100 μm) followed by Mira cloth (25 μm) repeatedly to separate the rough debris. The temperature and pH of the retting water during the retting season was 25–30 °C and 5.04–7.75, respectively.

### Isolation and purification of bacterial strains

The composite samples were diluted up to 10^−5^ in peptone water (Peptone 10 g/L, NaCl 5 g/L; pH 7.2 ± 0.2). Then 100 μl of samples from each dilution were spread on Nutrient agar (NA) (Peptone 5 g/L, Yeast extract 2 g/L, Meat extract 1 g/L, NaCl 5 g/L, Agar 15 g/L; pH 7.2 ± 0.2) and incubated at 34 °C for 24 hour in aerobic and anaerobic condition (AnaeroGen™ AN0035 kit). All the media were collected from Sigma-Aldrich. Triplicate set of plates were used for all the treatments throughout the study. Bacterial colonies were picked up based on different appearance and colony characteristics were determined according to Holtz^40^.

### Enzyme activity of the isolates

#### Solid culture extra-cellular enzyme assay

Primary screening of the isolates having pectinase, xylanase and cellulase activities was done by single touch inoculation of each colony onto 0.5% pectin, xylan and Carboxymethyl cellulose (CMC) agar respectively and incubated at 34 °C for 24 hours. Plates were observed for enzyme activity by flooding them with iodine solution (Iodine 1 g and Potassium Iodide 5 g for 330 ml iodine solution)^41^. Presence of clear zone developed through hydrolysis around the growth indicated positive enzyme activity and the extent of clear zone indicated the capacity.

#### Liquid culture extra-cellular enzyme assay

Bacteria were cultured in 100 ml Nutrient Broth (Peptone 5 g/L, Yeast extract 2 g/L, Meat extract 1 g/L, NaCl 5 g/L; pH 7.2 ± 0.2) and incubated at 34 °C in shaking incubator with 200 rpm for 20 hours. After incubation, a suitable volume of bacterial cultures were centrifuged at 22,000 × g rpm for 20 min and filtered the supernatant through Millipore filter (0.22 μm). The filtrate was subjected to the screening of three (pectinase, xylanase and cellulose) enzymatic activities by cup-plate diffusion technique^42^. The 4.0 mm diameter wells were prepared in agar plates containing pectin, xylan and CMC with a sterile cork borer to inoculate 25 μl of filtrate. After 24 hours of incubation at 34 °C, the plates were flooded with iodine solution. Presence of clear zone around the well indicated the enzymatic activity of the isolates. The average diameter (mm) of clear zones of 4 replications for each isolate was recorded.

#### Quantitative extra-cellular enzyme assay

Quantitative enzyme assay of retting isolates was based on the determination of reducing sugars produced as a result of enzymatic hydrolysis of pectin, xylan and cellulose by dinitrosalicylic acid reagent (DNS) method^43^. For the assay, 5 ml bacterial culture grown in modified MS medium containing 0.3% KH_2_PO_4_, 0.6% Na_2_HPO_4_, 0.2% NH_4_Cl, 0.5% NaCl, 0.01% MgSO_4.7_H_2_O supplemented with 0.5% yeast extract and 0.5% pectin (for pectinase production) or xylan (for xylanase production) or carboxymethyl cellulose (for cellulase production) was centrifuged at 10,000 rpm for 10 min at 4 °C and the clear supernatant was used as crude enzyme. Beechwood xylan 1% and carboxymethyl cellulose (CMC) 1% (Sigma-Aldrich) were prepared in 0.05M Na-citrate buffer of pH 5.3 and pH 4.8 respectively. Whereas Polygalacturonic acid 0.5% was prepared in 0.1M phosphate buffer (pH 7.5) and used as substrates for xylanase, cellulase and pectinase. The reaction mixture contained 900 μl of respective substrate (pectin and xylan) and 100 μl of appropriately diluted enzyme (pectinase, and xylanase), whereas, 100 μl of distilled water was used for enzyme blank. In case of cellulase, 1 ml of substrate (CMC) and 1 ml of appropriately diluted crude enzyme were used and were incubated at 50 °C for 10 min and the reaction was terminated by adding 1 mL of 3,5-dinitrosalicylic acid (DNS) and boiling (92 °C) for 5 min. Finally, the tubes were cooled and optical density (OD) was measured using spectrophotometer (Bio-Rad SmartSpec Plus) at 540 nm. For pectin, xylan and CMC, reducing sugar concentrations were estimated as D-galacturonic acid D-xylose and D-glucose equivalents, using a calibration curve constructed with D-galacturonic acid (Sigma-Aldrich, ≥98.0% purity) D-xylose (Sigma-Aldrich, ≥99% purity) and D-glucose (Sigma-Aldrich, ≥99.5% purity) respectively. The enzyme unit (U) was defined as the amount of enzyme that catalyzes the formation of 1 μmol of reducing sugar per minute under the specified assay conditions.

#### Isolation of genomic DNA

Genomic DNA was isolated following the method of Sambrook and Russel^44^ and treated with RNase A. The quantity and purity of DNA was assessed using a NanoDrop 2000 spectrophotometer (NanoDrop, Thermo Scientific). The integrity of DNA was evaluated by 1% agarose gel electrophoresis.

#### Amplification of 16S rDNA and PCR product purification

PCR amplification of 16S rDNA gene was carried out in GeneAmp PCR System 9700 (Applied Biosystems, Life Technologies, USA) by using forward primer 27 F (5′-AGAGTTTGATCMTGGCTCAG-3′) and the reverse primer 1492 R (5′-GGTTACCTTGTTACGACTT-3′). Final volume of PCR mixture was 50 μl containing 5 μl of 50 ng/μl genomic DNA, 0.25 μl of Platinum Taq DNA Polymerase (Invitrogen, Life Technologies, USA), 5 μl of 10x PCR buffer with 2 μl of 20 mM MgSO_4_, 1 μl of 10 mM dNTP (Invitrogen, Life Technologies, USA), 2 μl Dimethyl sulfoxide (DMSO), 2 μl of 10 μM each primer and 32.75 μl of nuclease free water. The cycling parameter consisted of 25 cycles: denaturation at 94 °C, 30 s; primer annealing at 55 °C, 30 s; extension at 68 °C, 1 min. Before amplification cycle, DNA was denatured for 5 min at 94 °C and after amplification an extension step for 7 min at 68 °C was performed. All the amplified PCR products were eluted from agarose gel using Qiagen QuickSpin PCR purification columns (Qiagen,Catalog No. 28706).

#### Sequencing of 16S rDNA fragment, assembly and BLAST search

The amplified and purified PCR fragments were sequenced in ABI 3730XL DNA Analyzer (Applied Biosystems, Life Technologies, USA) with the primer 27F (5′-AGAGTTTGATCMTGGCTCAG-3′), 533R (5′-TTACCGCGGCTGCTGGCAC-3′), 981R (5′-GGGTTGCGCTCGTTGCGGG-3′) and 1492R (5′-GGTTACCTTGTTACGACTT-3′). Sequencing reaction was performed by using Big Dye V3.1 sequencing reagents (Applied Biosystems, Life Technologies, USA) following the manufacturer’s protocol. The four sequences for each sample were assembled using CAP3 Sequence Assembly Program, to obtain the full 16S rRNA genes. The 16S rDNA sequences of the isolated strains were compared to public databases available in NCBI. Identification to the species level was determined as a 16S rDNA sequence similarity of >99% with that of the prototype strain sequences in the GenBank.

#### Phylogenetic analysis

The 16S rDNA sequences of isolates were aligned with the MUSCLE and phylogenetic trees were inferred using the neighbor-joining method^45^. The software MEGA, version 6.0, was used to construct trees^46^. Bootstrap analysis (100 replicates) was used to test the topology of the neighbor-joining method.

#### Extracellular enzymatic assay of bacterial consortia

A total of 167 consortia of different combinations containing 3 to 10 bacterial isolates, were selected to test synergistic effects in extracellular enzyme production. For each treatment, primary inoculum of individual strain was prepared by growing in 20 ml NB at 30 °C for 24 hours which was used to inoculate 99 ml NB in 1:99 ratio after adjustment of Optical Density (OD) at 6–7(±0.4) (A = 600). After attaining mid-log phase (0.4–0.6 OD) of growth, bacterial cultures were mixed equally and OD of mixed culture was adjusted at 6.5–7.0 to inoculate 99 ml NB in 1:99 ratio and incubated for their respective incubation period (Optimization). Enzyme activity was checked according to same procedure as extracellular enzyme assay. The bacterial isolates were further screened for enzyme activity by quantitative assay. To study the pectinase, xylanase and cellulase activities in various consortia, the respective enzyme producing media were simultaneously inoculated with the bacterial strains and the cell free supernatants were used for the assays. The enzyme producing media composition and quantitative enzyme assay procedure were as same as described above in Quantitative extra-cellular enzyme assay section.

### Retting efficiency test

#### Small scale

Artificial plastic retting tanks (Length 150 inch, width 14 inch and depth 7 inch) were used for jute retting trial. Green ribbons were extracted from 120 days aged defoliated jute plant (*Corchorus olitorius*, variety O-4) with an average height 3.30 m and diameter 23 mm by a ribbon extractor machine (CRIJAF, 2011). Two kg of green ribbon was submerged in 10 L of distilled water at a substrate liquor ratio of 1:5. Three consortia C-51, C-67 and C-90 with 5, 6 and 7 bacterial strains respectively were used as inocula in each treatment. The bacterial inocula were added with water of 10:1 at 1 × 10^8^ cells/ml. Plastic tanks were covered with cork sheet and kept at green house to maintain the temperature at 30–32 °C until completion of retting. Each treatment was carried out in three replicated tanks. A positive and negative control was maintained using 10 L natural river water and 10 L distilled water without inocula, respectively.

#### Large scale

Large scale retting experiment was also carried out with 10 kg and 50 kg of 120 days aged ribbon in cemented retting concrete tank. We have used combination C-67 containing 6 bacterial isolates that produced best results in plastic tank retting experiment. Ribbon and inocula and water were added in 1:1:5. Number of days required for retting, average day temperature, OD and pH of retting liquor during progress of retting was periodically recorded. After completion of retting, fibre was extracted, washed and sun dried.

#### Fibre quality test method

Bundle strength of jute fibre was determined by taking 20 randomly selected jute samples in each set and following the method described by Bandyopadhyay and Mokhapadhyay^47^ on a Pressly fibre bundle strength tester (Model-215, USA). 100 reading from each set of yarn were taken to measure Twisting force and luster of the fibre on a Goodbrand Manual Twist Tester (Goodbrand and Company Ltd, UK 80600) and Reflection meter (Model-577, USA) respectively.

### Statistical analysis

All experiments were laid out a complete randomized design with four replications. Analysis of variance and comparison of means were calculated with the statistical package Mstat-C v2.10. Means were compared by using the Least Significance Differences (LSD) test (*P* < 0.05).

## Supplementary information

Supplementary Figures.

Supplementary Table 1.

Supplementary Table 2.

Supplementary Table 3.

Supplementary Table 4.

## Data Availability

All 16S rRNA partial gene sequences are publicly available from the GenBank under the accession number MH010052 to MH010195, with specific numbers listed in Supplementary Table 3.
