# Supplementary material for: Pectinolytic Bacterial Consortia Reduce Jute Retting Period and Improve Fibre Quality
Source: Sci Rep. 2020 Mar 20;10:5174. doi: 10.1038/s41598-020-61898-z (PMC7083874; doi:10.1038/s41598-020-61898-z)
Supplement: Supplementary file 1 — Supplementary Figures. [file 41598_2020_61898_MOESM1_ESM.docx]

**Pectinolytic Bacterial Consortia Reduce Jute Retting Period and Improve Fibre Quality**

Rajnee Hasan^1†^, Nasima Aktar^1†^, Shah Md. Tamim Kabir^1^, Ummay Honi^1^, Abdul Halim^23^, Rahin Islam^4^, Muhammad Delwar Hossain Sarker^1^, Md. Samiul Haque^13^, Md. Monjurul Alam^13^, Md. Shahidul Islam^13*^

^1^Basic and Applied Research on Jute Project, Bangladesh Jute Research Institute, Dhaka, Bangladesh

^2^McMaster University, Hamilton, Canada

^3^Bangladesh Jute Research Institute, Dhaka, Bangladesh

^4^Eskayef Pharmaceuticals Limited, Bangladesh

^†^ Contributed equally to the study


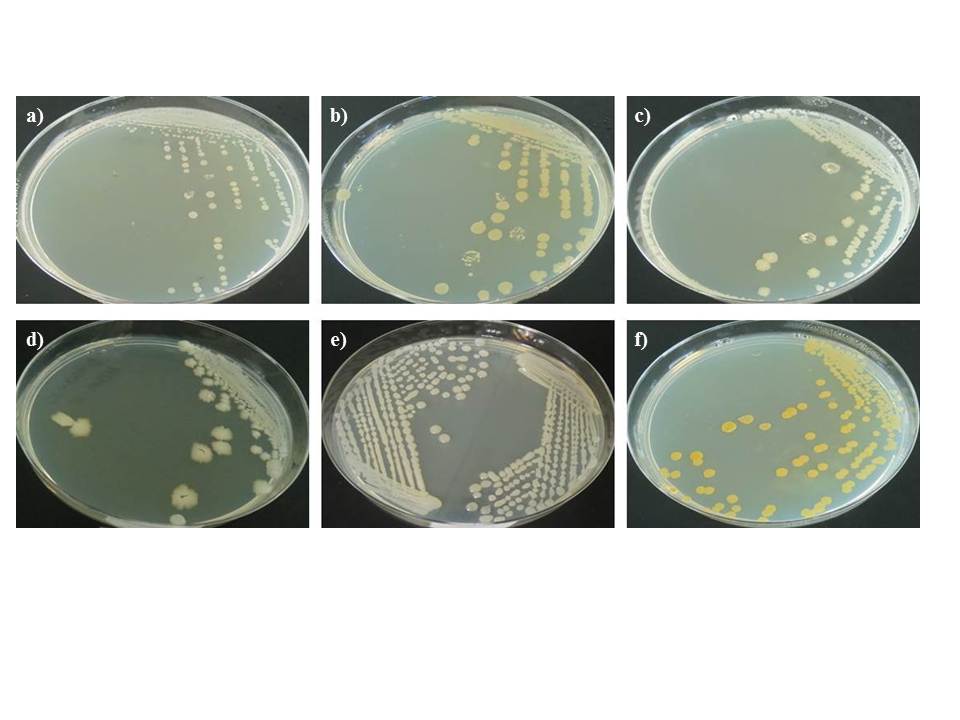


**Figure S1.** Different colony morphology of the isolates - shape, color, margin, evaluation, size, opacity and appearance of the isolates are given respectively. (a) Circular, off white, wooly, convex, small, opaque and dull; (b) Circular, cream off white, entire, flat, medium, opaque and dull; (c) Irregular, off white, undulate, flat with raised center, medium, opaque and dull; (d) Irregular, off white, lobate, raised, medium to large, opaque and dull; (e) Circular, off white, entire, convex, small to medium, opaque and Glistening; (f) Circular, orange, entire, raised, small to medium, opaque and dull.


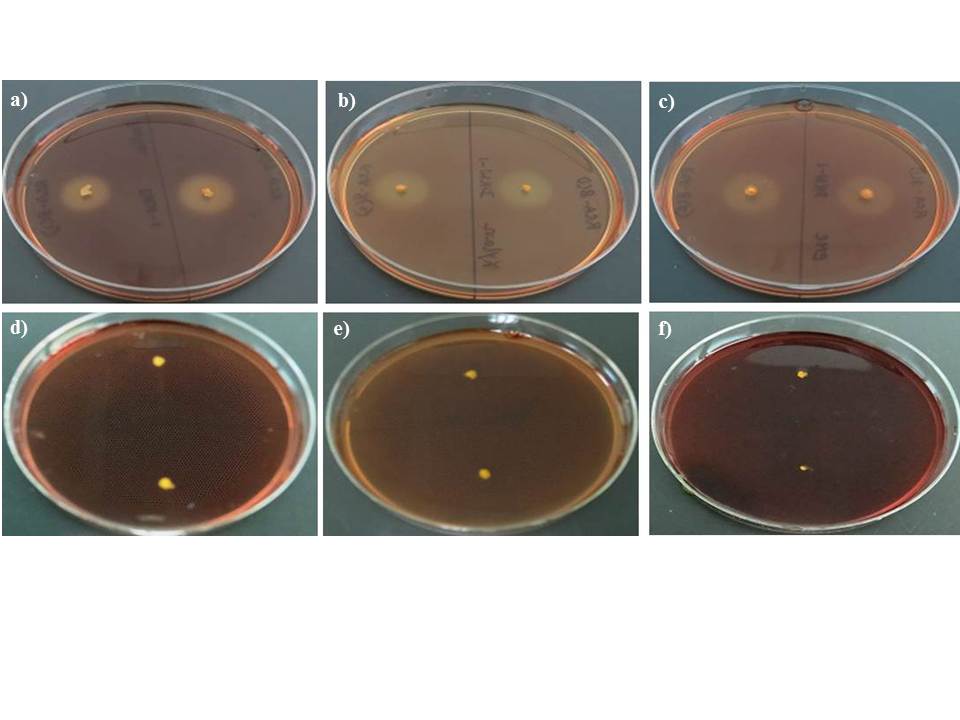
**Figure S2.** Screening of isolates by culturing on pectin, xylan and cellulose containing plate. (a), (b), (c) shows pectinase, xylanase and cellulase activity respectively. (d), (e), (f) shows no enzymatic activity.


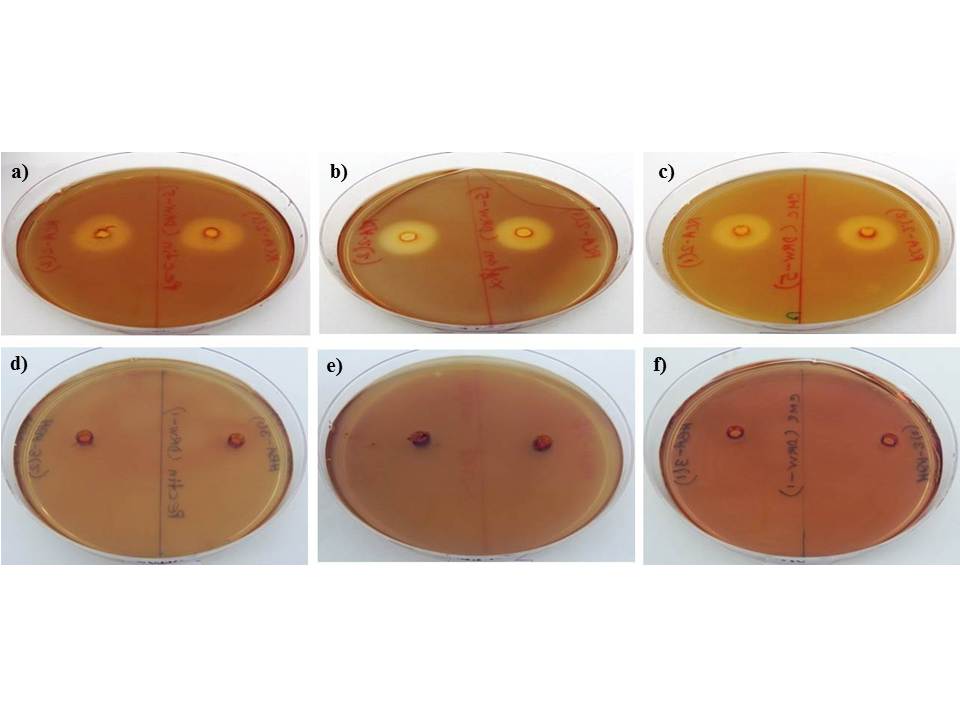
**Figure S3.** Screening of isolates by extra-cellular pectinase, xylanase and cellulase enzyme activity in specific substrate plate. (a), (b), (c) shows positive activity of pectinase, xylanase and cellulose respectively. (d), (e), (f) shows no enzymatic activity.
